# Multiple capsid-stabilizing interactions revealed in a high-resolution structure of an emerging picornavirus causing neonatal sepsis

**DOI:** 10.1038/ncomms11387

**Published:** 2016-07-20

**Authors:** Shabih Shakeel, Brenda M. Westerhuis, Ausra Domanska, Roman I. Koning, Rishi Matadeen, Abraham J. Koster, Arjen Q. Bakker, Tim Beaumont, Katja C. Wolthers, Sarah J. Butcher

**Affiliations:** 1 Institute of Biotechnology and Department of Biological Sciences, Viikinkaari 1, 00014 University of Helsinki, Finland; 2 Department of Medical Microbiology, Laboratory of Clinical Virology, Academic Medical Center, Meibergdreef 9, 1105 Amsterdam, The Netherlands; 3 Leiden University Medical Center, Department of Cell Biology, Postal Zone S1-P, P.O. Box 9600, 2300 RC Leiden, The Netherlands; 4 Netherlands Centre for Electron Nanoscopy, Institute of Biology, Ensteinweg 55, 2333 RC Leiden, The Netherlands; 5 AIMM Therapeutics, Academic Medical Center, Meibergdreef 59, 1105 BA Amsterdam, The Netherlands

## Abstract

The poorly studied picornavirus, human parechovirus 3 (HPeV3) causes neonatal sepsis with no therapies available. Our 4.3-Å resolution structure of HPeV3 on its own and at 15 Å resolution in complex with human monoclonal antibody Fabs demonstrates the expected picornavirus capsid structure with three distinct features. First, 25% of the HPeV3 RNA genome in 60 sites is highly ordered as confirmed by asymmetric reconstruction, and interacts with conserved regions of the capsid proteins VP1 and VP3. Second, the VP0 N terminus stabilizes the capsid inner surface, in contrast to other picornaviruses where on expulsion as VP4, it forms an RNA translocation channel. Last, VP1's hydrophobic pocket, the binding site for the antipicornaviral drug, pleconaril, is blocked and thus inappropriate for antiviral development. Together, these results suggest a direction for development of neutralizing antibodies, antiviral drugs based on targeting the RNA–protein interactions and dissection of virus assembly on the basis of RNA nucleation.

## Abstract (web summary)

Human parechovirus 3 (HPeV3) can cause severe central nervous system infections and is a major cause of neonatal sepsis. Here the authors determine the structure of HPeV3 that provides a high-resolution view of the capsid's organization and shows multiple interactions of the RNA genome with coat proteins.

## Graphical abstract

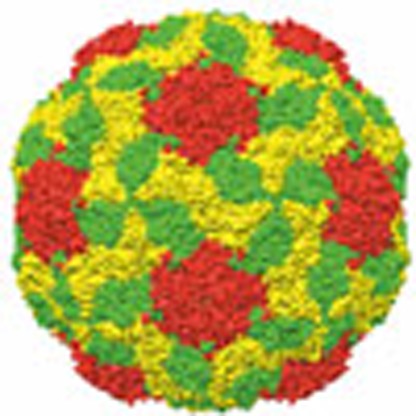

The Picornaviridae is a family of small, icosahedrally-symmetric, positive-sense, single-stranded RNA viruses. *Parechovirus A* is a species within this family with 16 genotypes and it is mainly associated with mild infections in humans especially children. However, an emerging pathogen, human parechovirus 3 (HPeV3) can cause severe central nervous system infections such as meningitis^1^, and is a leading cause of neonatal sepsis^2^. There are no antivirals or vaccines available to combat HPeV infection. Unlike many other picornaviruses, HPeV are characterized poorly both in terms of structure and function, except for HPeV1 where the receptor is known^3^. The great differences in tropism shown by HPeV3 compared to the other HPeV, makes it essential to investigate HPeV3 structural properties for a better understanding of its pathogenesis and potential receptor binding.

We utilized cryo-electron microscopy and image reconstruction to analyse the structure of HPeV3 on its own and in complex with a human monoclonal antibody Fab. The virion structure shows that VP1 pocket-binding drugs, such as pleconaril, are unlikely to bind to HPeV; that VP0 is an important protein for stabilizing the inner surface of the capsid, and finally, that the assembly of HPeV is most likely controlled by multiple interactions of the genome with the capsid, through conserved amino acids in VP1 and VP3 and stem-loop structures in the RNA. We isolated and characterized an HPeV3-specific human monoclonal antibody, which could be very useful for advancing virus diagnostics and studying virus–host interactions.

## Results and Discussion

### HPeV3 structure

**Figure 1:**
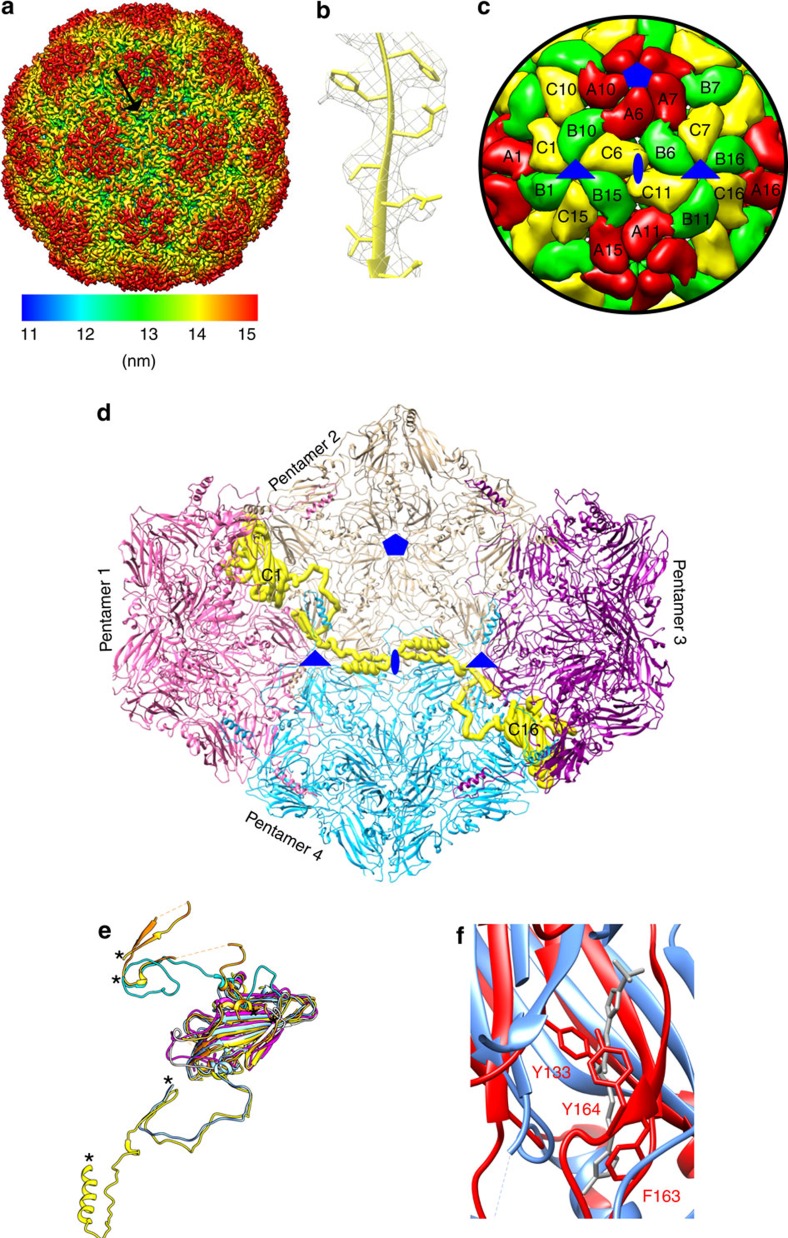
HPeV3 structure. (**a**) Radially coloured isosurface representation down a twofold axis of symmetry of HPeV3 at 4.3 Å resolution shown at 3σ above the mean threshold. The arrow indicates the canyon. Colour key shows the radial colouring from the centre of the virus in nm. (**b**) Representative fit of VP0 atomic model to electron density (mesh). (**c**) A zoomed-in view of the capsid model showing the positions of VP0 (yellow), VP1 (red) and VP3 (green) in a *T*=1 (pseudo *T*=3) arrangement. The symmetry axes are marked in blue (fivefold pentagon, threefold triangle, twofold ellipse). The capsid is made from 12 pentamers of VP0, VP1 and VP3. Some of the proteins in neighbouring pentamers are marked (VP0, C1-C16; VP1, A1-A16; VP3, B1-B16). (**d**) A stabilizing network of VP0 N-terminal arms traverses the inner side of the capsid. The path of one N terminus is highlighted in yellow (C1) from pentamer 1 (pink) travelling via VP3 (gold) of pentamer 2 (gold) to interact with the N terminus of C16 from pentamer 3. These VP0 N termini obstruct the pore at the twofold symmetry axis between pentamer 2 and pentamer 4. (**e**) Unusual position of HPeV3 VP0 N terminus compared to other picornavirus is shown by superposition of VP0 from HPeV3 (yellow), with poliovirus (1pov; orange), EV71 (3vbu, magenta) and HAV (4qpg, blue). In addition, the locations of VP4 (cyan) and VP2 (white) of EV71 (3vbf) in comparison to HPeV3 VP0 are also shown. N termini for all the superimposed proteins are marked with asterisks in **e**). (**f**) HPeV3 VP1 β-barrel region is shown superimposed on a pocket factor containing EV-D68 VP1 (4wm7; VP1, blue; pocket factor, grey). The HPeV3 hydrophobic pocket is blocked by residues Y133, F163 and Y164.

The HPeV3 virus preparations were free of empty capsids as we have observed previously for HPeV1 (ref. 3). We determined a 4.3 Å resolution HPeV3 structure using electron cryo-microscopy and single particle analysis (Fig. 1a; Supplementary Table 1; Supplementary Fig. 1). Homology models of capsid proteins VP0, VP1 and VP3 were used as starting models to generate an atomic model of HPeV3 constrained by the density from the reconstruction (Fig. 1b–d and Supplementary Movie 1). The HPeV3 capsid is composed of 60 copies of three β-jellyroll proteins, VP0, VP1 and VP3 in a *T*=1, pseudo *T*=3 arrangement (Fig. 1c and Supplementary Movie 1). Unusually for a picornavirus, we showed by polyacrylamide gel electrophoresis protein analysis and from the structure, that VP0 does not undergo autocatalytic cleavage into VP4 (N-terminal) and VP2 (C-terminal) in the virion (Fig. 1 and Supplementary Fig. 2). Similar to many picornaviruses^4^, HPeV3 possesses an open channel at each fivefold vertex^5^ (Supplementary Fig. 3) and a canyon for potential receptor and antibody binding, however, it is wide and shallow (Fig. 1a). Five VP3 N termini come together to form an annulus at the base of the channel, giving intra-pentamer stability (Supplementary Fig. 3). Picornaviral capsid channels have been implicated in RNA genome release that is initiated by receptor binding, and the exposure of VP4 and the VP1 N terminus through conformational change^5^,^6^. RNA uncoating in parechoviruses may exploit a different mechanism. In contrast to VP4 from enteroviruses, the equivalent VP0 N terminus does not surround the annulus ready for release^4^,^7^. Instead the VP0 N terminus straddles an adjacent threefold on the inner surface of the capsid, forming a loop that stabilises the twofold-related VP0 from another pentamer (Fig. 1d, pentamers 1 and 3), a conformation that has not been previously observed to our knowledge (Fig. 1e). This sets HPeV apart from other picornaviruses and from the otherwise closely related Ljungan virus (*Parechovirus B*) infecting voles^8^. This interaction of the symmetry-related VP0 N termini at the twofold across neighbouring pentamers lies beneath two interacting VP0 hA1 α-helices. During RNA uncoating in many picornaviruses, these hA1 helices move apart^9^, resulting in extrusion of the amphipathic helix-carrying VP1 N terminus, which lodges itself into the cell membrane and the expulsion of VP4 to form a channel for RNA release. In HPeV, the VP0 N terminus may be the one which is first extruded out of the capsid.

### Ordered-structure of HPeV3 RNA

**Figure 2:**
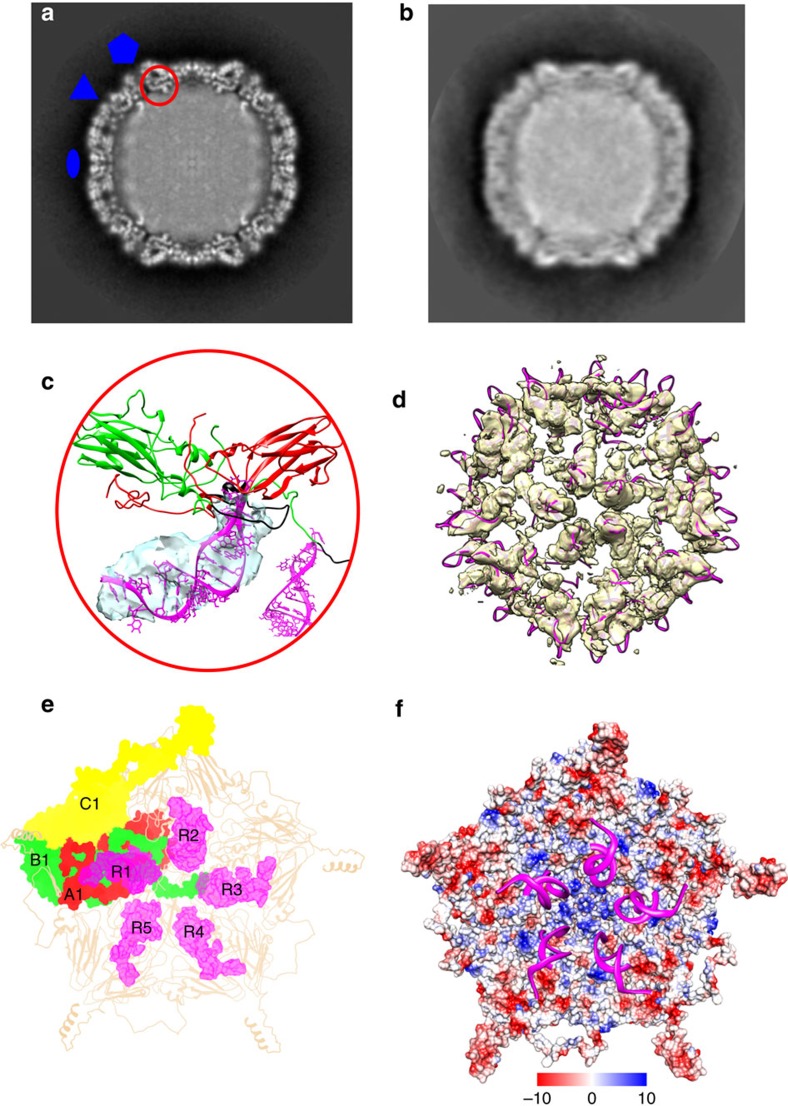
Ordered RNA–protein interactions. (**a**) Central cross-section of the unfiltered HPeV3 icosahedrally-symmetric reconstruction with three symmetry axes marked. The red circle in (**a**) and (**c**) indicates one of the regions where the capsid proteins interact with finger-like RNA densities. (**b**) Central cross-section of the unfiltered HPeV3 asymmetric reconstruction. (**c**) Enlargement of the VP3 (green) and VP1 (red) atomic model in intimate contact with an RNA model (magenta, R1) within its asymmetric unit. The VP3N terminus also interacts with a neighbouring RNA molecule within the pentamer. The fit of one of the RNA models in the RNA EM density (transparent isosurface) is shown. The RNA interacting regions in VP1 and VP3 are coloured black. (**d**) The icosahedrally-symmetric copies of the fitted-RNA model from **c** shown in the HPeV3 asymmetric reconstruction's RNA density (yellow transparent surface shown at 2σ above the mean threshold). (**e**) VP1 and VP3 interaction with the RNA is shown in the context of the inner surface of a pentamer. The N terminus of VP3 (B1) and regions of VP1 (A1) interact with the RNA (R1) within its asymmetric unit and also with an RNA (R3) from the next-but-one asymmetric unit within a pentamer. The proteins are marked as in Fig. 1c. The RNA models are marked R1-R5 for their respective asymmetric units as in Fig. 1c). (**f**) The inner surface of a pentamer of the HPeV3 model shown as an electrostatic potential surface with the conserved RNA motif (magenta) shown in ribbon. The scale for the charge distribution is also shown. The RNA interaction with the capsid protein does not appear to be driven by electrostatics as the interacting region on the capsid proteins appear to be a mix of positive (blue), negative (red) and neutral charges (white).

**Figure 3:**
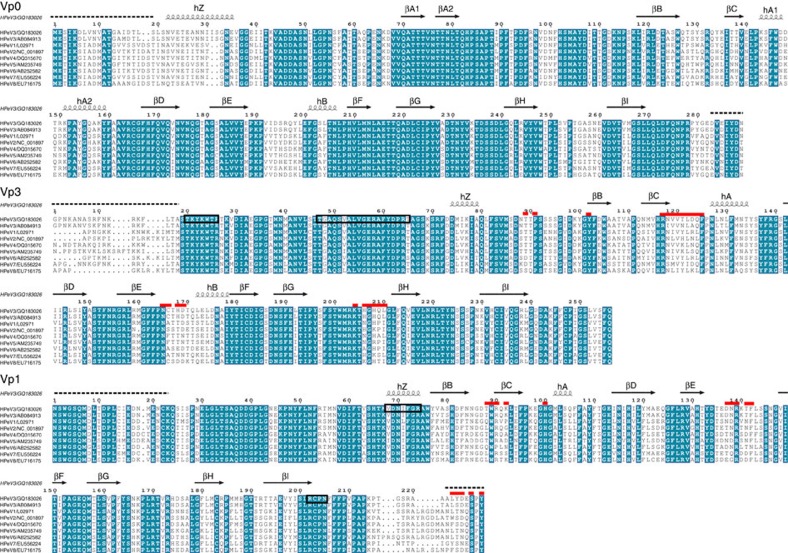
Structure-based sequence alignment of human parechoviruses. Multiple sequence alignment of HPeV1-8 amino acid sequences of capsid proteins VP0, VP3 and VP1 using protein BLAST^45^ is shown. The secondary structure elements from the atomic model of HPeV3 152037 are shown above the alignment as α-helices (spirals), β-sheets (arrows), disordered regions (dashed lines). Sequence annotations on the left correspond to the virus genotype/GenBank IDs. Sequence identity among all the aligned human parechoviruses (blue highlight), RNA binding sites (black boxes) and conformational epitope for mAb AT12-015 (red lines) are indicated on the HPeV3 isolate 152037 protein sequences. Disordered regions of the structural proteins were truncated from the atomic HPeV3 model. The disordered VP1 C terminus is exposed on the outside of the capsid and was identified in the footprint of Fab AT12-015 by comparison to other picornavirus structures. The footprint incorporates four amino acid differences between HPeV3 152037 and A308/99 in the variable surface loop VP3 βChA and the C terminus of VP1. The amino acid sequence identity of 19 P1 polyproteins from all HPeV3 isolates available in GenBank is 99%.

Unusually, about 25% of the genomic RNA is highly ordered, and at high occupancy in the reconstruction. Finger-like RNA densities come into close contact with capsid proteins VP1 and VP3 around the vertices (Fig. 2a) that could accommodate about 30 nucleotides. The sequence of the RNA was not evident even though the RNA density was at the same resolution as the protein density (Supplementary Fig. 1c). This suggested repetition of a structural rather than a long sequential RNA motif, and indeed we did not find 60 identical, 30 residue-long repeating sequences in the genome. However, the 3′-end of the genome has previously been predicted to be highly structured^10^,^11^. We modelled an RNA stem-loop model containing residues 7,181–7,210 from the 3′-end of the genome (Genbank ID GQ183026) into the density with a cross-correlation value of 0.86. To further confirm that all the 60 symmetry-related sites have high RNA occupancy, we performed an asymmetric reconstruction of the large data set of HPeV3 (41,845 particles). We were still able to identify the ordered RNA at all 60 sites albeit at variable signal intensity. The RNA model fitted into all the 60 sites with cross-correlations ranging from 0.86 to 0.88 (Fig. 2b,d, Supplementary Fig. 1d and Supplementary Movie 1). Moreover, we identified the conserved regions in the N terminus of VP3 (T20-R26, T47-T65), hZ helix and the βIC terminus regions of VP1 (R202-N205) as the structured RNA interaction sites (Fig. 2c,e; Fig. 3). We mapped the distribution of charge on the inner surface of the capsid, and noticed that although the surface is not highly positively charged, unlike in Ljungan virus^8^, there are a few conserved basic residues in these regions from all *Parechovirus A*, notably, K21, K23, R58 and R64 in VP3, VP1, R74 and R202, respectively (Figs 2f and 3). As the VP3 N terminus forms the annulus, it also interacts with two RNA stem-loops (Fig. 2e). Taken together, these results indicate that the co-assembly of the HPeV capsid proteins with the viral genome may be dictated both by electrostatic interactions, and by a cooperative effect between repeated elements of secondary structure (packaging sequences) throughout the genome, recognized by the capsid proteins. In practice, the RNA genome could nucleate capsid assembly at multiple sites. Each VP3 molecule thus incorporated, may help to condense the genome as it can bring together two stem-loops from disparate regions of the genome (Fig. 2c,e). This hypothesis is inspired by the work on the single-stranded RNA bacteriophage, MS2, where multiple packaging sequences have been identified that promote co-assembly with the capsid protein, providing a competitive advantage for viral RNA packaging over host RNA during the initial stages of assembly^12^. These RNA–protein associations could be exploited for designing specific antivirals against HPeV3 to interfere with the association.

### VP1 hydrophobic pocket

The antiviral drug, pleconaril and its derivatives usually block enteroviruses infections such as EV-D68 (ref. 4). These drugs expel the lipid (‘pocket factor') present in the hydrophobic pocket of the VP1 β-barrel and block RNA uncoating by stabilizing the capsid^4^. The channel to the hydrophobic pocket in HPeV3 VP1 is blocked by three large, conserved side chains Y133, F163 and Y164 (Figs 1f and 3) compared to EV-D68 (ref. 4). Thus, we can explain observations that pleconaril does not work in the clinic^2^, and predict that such pocket-factor mimics will not work against any of the HPeV so far sequenced.

### HPeV3–Fab complex structure

**Figure 4:**
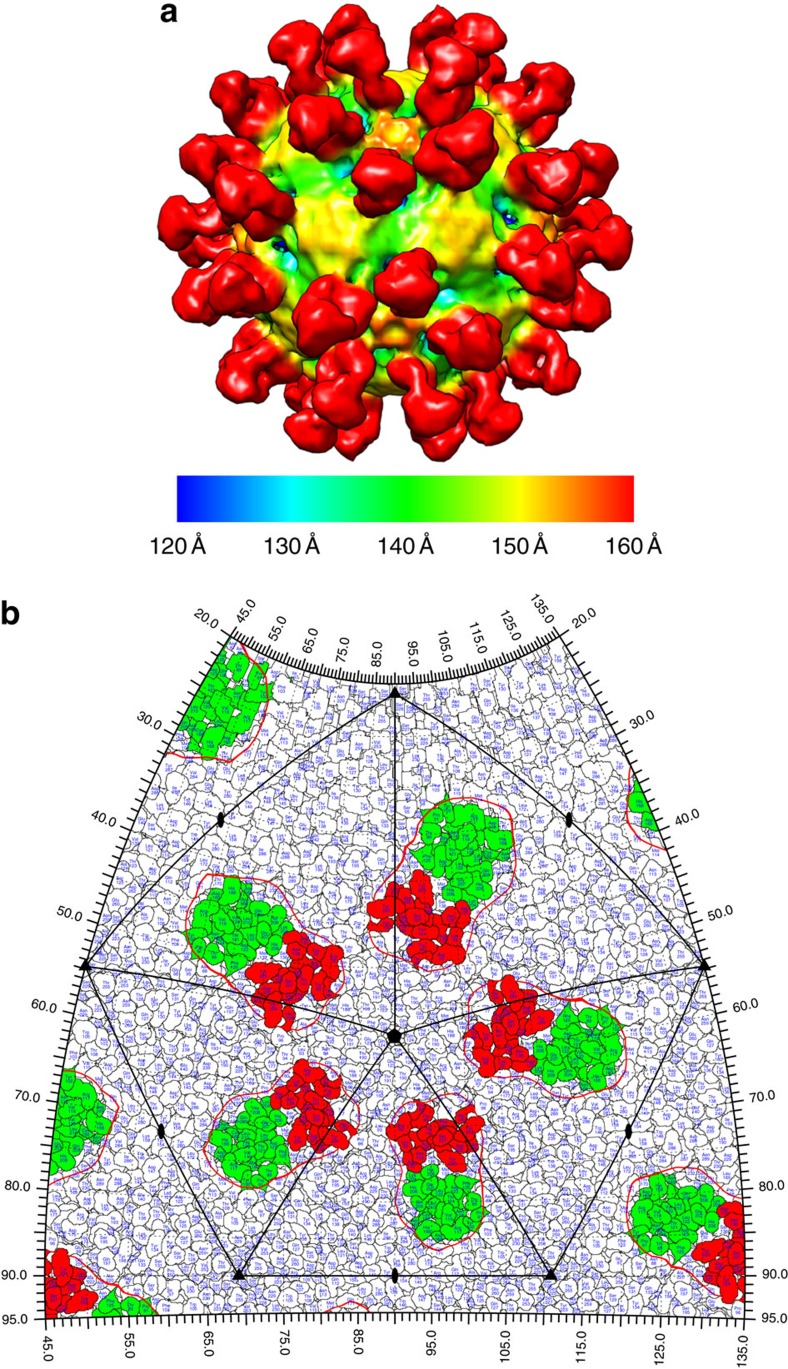
HPeV3–Fab structure. (**a**) Radially coloured isosurface representation of HPeV3–Fab AT12-015 complex at 15 Å resolution shown at 1.5σ above the mean threshold. The Fab molecules (red) bind around the canyon region. (**b**) HPeV3 roadmap. The Fab footprint (red contour) is mapped to VP3 (green) and VP1 (red).

We isolated an HPeV3-specific human monoclonal antibody, AT12-015 that bound to HPeV3 isolates 152037, A308/99 and two clinical isolates in infected cells. It did not neutralize 152037 in a Vero cell-line-based assay. We solved the structure of HPeV3 isolate 152037 in complex with Fab AT12-015 (Supplementary Table 1; Fig. 4a; Supplementary Fig. 1). The Fab molecules recognize a conformational epitope on the rim of the canyon (Fig. 4a). Contributions come from regions in both VP3 (hZβB, βChA, βEβF and βGβH loops) and VP1 (βBβC, βCβD and βEβF loops, C terminus) mainly conserved in HPeV3 explaining the antibody's specificity to this genotype (Fig. 3). The Fab footprint encompasses the VP1 C terminus where many other HPeV contain an RGD-motif to bind their integrin receptors (Figs 3 and 4b)^3^. As AT12-015 is specific for HPeV3 and did not bind to any of the other HPeV genotypes tested it could be very useful for advancing virus diagnostics and studying virus–host interactions. Further comparison with epitopes from neutralizing patient sera could help us understand the mechanism of neutralization in patients^13^.

Overall, this work shows multiple, stabilizing RNA–capsid interactions as a novel target for anti-parechovirus drugs. We revealed the unusual stability of these capsids at the intra-pentamer level by VP3 interactions and at the inter-pentamer by VP0, and propose that the uncoating mechanism of the HPeV genome will be significantly different to that of other picornaviruses.

## Methods

### Virus purification and inactivation

The HPeV3 isolate 152037 was grown on an African green monkey (Vero) cell line maintained in Dulbecos Modified Eagles Medium supplemented with glutamax (1 × ), non-essential amino acids (1 × ), streptomycin (0.1 μg ml^−1^), penicillin (0.1 μg ml^−1^) and 10% heat-inactivated foetal bovine serum (FBS). Confluent cell layers (90%) were inoculated with HPeV3 isolate 152037 at a 0.01 multiplicity of infection in medium containing 2% FBS. At 100% infection of the cell monolayer evident by the cytopathic effect (CPE), the cells and spent media were collected, freeze-thawed three times and clarified by low speed centrifugation^14^. The supernatant was filtered and concentrated by ultrafiltration in Centricon 100 kDa devices (Millipore). The resulting preparation was purified by differential ultracentrifugation on a CsCl step gradient where the top density was 1.2502, g cm^−3^ and the bottom 1.481 g cm^−3^ CsCl (32,000*g*, Beckmann SW41 Ti rotor, 18 h at 4 °C). The virus band was buffer-exchanged with 10 mM Tris–HCl, pH 7.5, 150 mM NaCl, 1 mM MgCl_2_ (1 × TNM buffer) using ultrafiltration in Centricon 100 kDa devices (Millipore) to remove CsCl. The ultracentrifugation and buffer exchange was repeated a second time. The purified HPeV3 isolate 152037 was inactivated by adding 0.1 mg ml^−1^ formaldehyde and keeping at 37 °C for 72 h. Inactivation was confirmed by infecting Vero cells with inactivated virus. No CPE was seen up to 7 days.

HPeV1-Harris and HPeV2-2008 were provided by the Dutch National Institute for Public Health and Environment, Bilthoven, the Netherlands. HPeV4-251176, HPeV5-552322, HPeV6-550389, and two HPeV3 clinical isolates were obtained from the Laboratory of Clinical Virology, Academic Medical Center, Amsterdam, the Netherlands. For virus stocks HPeV1, HPeV2, HPeV3 A308/99 (kind gift from Hiroyuki Shimizu and Miyabe Ito) and HPeV4-6 were grown on a HT29 cell line in Eagle's minimum essential medium with L-glutamic acid (0.2 × ), non-essential amino acids (1 × ), streptomycin (0.1 μg ml^−1^), penicillin (0.1 μg ml^−1^) and 2% heat-inactivated FBS (HT29 cells were maintained in medium containing 8% heat-inactivated FBS). These strains were used in antibody AT12-015 binding and neutralization assays.

### Generation of antibodies and preparation of Fab fragments

The human monoclonal antibody AT12-015 against HPeV3 isolate 152037 was prepared from human blood^15^. Briefly, human memory CD27+IgG+ B cells were cultured using the AIMSelect method from healthy donors who had recovered from a confirmed HPeV3 isolate 152037 infection 1 year earlier^15^. Single cells were subcloned from B cell cultures where the supernatant showed binding to HPeV3-infected cells by immunofluorescence. RNA was isolated from the monoclonal B cells to retrieve the antibody heavy and light chain sequences. Unique sequences were used to generate recombinant protein in 293 T cells. IgG1 antibodies were subsequently purified using HiTrap Protein A columns on an ÄKTA instrument (GE). Fab fragments from AT12-015 were produced using a Pierce Fab micro preparation kit according to the manufacturer's instructions to achieve full digestion of the antibody. The resulting Fab fragments were mixed with HPeV3 capsids at a molar ratio of 60 to 1 and incubated for 30 min at 37 °C in 1 × TNM to allow virus–Fab complex formation, prior to vitrification.

### HPeV neutralization assay

Neutralization of HPeV3 isolate 152037 by AT12-015 was tested by infecting Vero cells with pre-incubated virus (100 TCID50 units) with varying amounts of the antibody (0.03–7.5 μg ml^−1^) at 37 °C for 1 h. Infected cells were monitored for the appearance of CPE every 24 h for 7 days. At day 7, RNA was extracted from the supernatant using a total nucleic acid isolation kit with the MagnaPure LC instrument (Roche Diagnostics), reverse transcribed and cDNA (complementary DNA) was used to estimate viral copy number by real time PCR using an LC480 instrument (Roche Diagnostics)^16^. The capacity of AT12-015 to neutralize HPeV3 isolate 152037 was also tested on BGM (buffalo green monkey kidney), A549 (human colon adenocarcinoma) and Caco2 (human colon adenocarcinoma) cell monolayers. Furthermore, the cross-neutralization by AT12-015 against HPeV1, HPeV2, HPeV4, HPeV5 and HPeV6 was also tested in Vero cells. Immunofluorescence of HPeV1-6 infected cells using AT12-015 was also checked.

### Electron cryo-microscopy and image processing

The formaldehyde-inactivated purified HPeV3 was vitrified by applying 3 μl of the sample on Quantifoil R3.5/1 grids, blotting for 2 s at a relative humidity of 92% and plunging into liquid ethane using a Leica EM GP. The grids were examined in a Cs-corrected FEI Titan Krios transmission electron microscope at 300 keV. The images were recorded on a Falcon II detector under low dose conditions at a nominal magnification of 59,000 × with a sampling size of 1.14 Å per pixel. Seven frames per image were collected in counting mode using FEI EPU automated single particle acquisition software. The movie frames were initially aligned with each other using motioncorr software^17^ before further processing.

The contrast transfer function of each micrograph was estimated using CTFFIND3 (ref. 18) and aligned averaged images containing drift or astigmatism were discarded. Particles were picked from the averaged image of all the seven aligned frames of the movie using the programme ETHAN^19^ with a box size of 401 pixels and inspected by eye in the programme suite EMAN^20^. A random model generated from 150 particles was used as a starting model to initiate full orientation and origin determinations of the full set of particles using AUTO3DEM 4.04 (ref. 21). The final model from AUTO3DEM was used as an initial reference model filtered to 60 Å for processing 41,849 particles in Relion 1.3 (ref. 22). These particles were classified into 100 classes by two-dimensional classification and the best class of 41,845 particles was selected, resulting in a 5.9 Å resolution icosahedrally symmetric model. They were further classified into four classes by three-dimensional classification with icosahedral symmetry imposed, and the two best classes containing 8,889 particles were selected for further refinement. Individual particle movies were realigned in Relion with a running average window of 5 and standard deviation of 1 on translation. Each particle from all the aligned movie frames was B-factor weight-averaged using the particle polishing step in Relion to compensate for the radiation damage resulting in an increased signal to noise ratio. The running average window used was 5, with a standard deviation of 100 on particle distance. The final refinement between two independent datasets gave a resolution of 4.56 Å on the basis of the 0.143 criterion FSC. After masking, the final resolution was 4.3 Å. A B-factor correction of –164.4 Å^2^ was automatically estimated and applied^23^. A local resolution was also estimated for the reconstruction using ResMap^24^.

For the asymmetric reconstruction, the orientations of the best 41,845 particles were refined for a further 16 rounds using the three-dimensional auto-refine option in Relion without imposing any symmetry. The final refinement between two independent datasets gave a resolution of 10.36 Å on the basis of the 0.143 criterion FSC.

Aliquots of HPeV3–Fab AT12-015 complexes were vitrified on glow-discharged ultrathin carbon film containing holey carbon copper grids (TED PELLA) using a home-built guillotine. The grids were examined in a FEI Tecnai F20 transmission electron microscope at 200 keV using a Gatan 626 cryostage. The images were recorded on Kodak SO-163 films under low dose conditions at a nominal magnification of 50,000 × . Films were digitized on a Zeiss scanner (Photoscan) at a step size of 7 μm giving a pixel size of 1.4 Å per pixel. The contrast transfer function of each micrograph was estimated as described above. Particles were picked on 4 × binned micrographs using the programme RobEM^21^ with a box size of 101 pixel. A random starting model was generated from 150 particles using AUTO3DEM v 4.05.1 (ref. 21). The full data set was divided into two and the orientations and origins were determined from independent reconstructions generated by each set. The final resolution of 15 Å from 564 particles was achieved on the basis of the 0.143 FSC criterion.

### Model building

The structures of the three HPeV3 capsid proteins were predicted by multiple-template comparative modelling using the I-TASSER server^25^. The template structures for VP0 included a user-defined template containing a fused structure of human enterovirus VP4 (PDB ID: 3vbf) N terminal to empty human enterovirus VP2 (PDB ID: 3vbo)^26^, as well as the automatically selected foot and mouth disease virus (PDB ID: 1qqp)^27^, poliovirus 1 (PDB ID: 1pov)^28^, bovine enterovirus (PDB ID: 1bev)^29^, echovirus 1 (PDB ID: 1ev1) (ref. 30) and hepatitis A virus (PDB ID: 4qpg)^31^. The C-score for the best model was –1.03.

For VP3, a user-provided template of empty human enterovirus (PDB ID: 3vbo)^26^ was used in addition to the following templates selected by the programme: coxsackievirus A9 (PDB ID: 1d4m)^32^, echovirus 1 (PDB ID: 1ev1) (ref. 30), human enterovirus (PDB ID: 3vbf)^26^, human enterovirus (PDB ID: 3vbh)^26^, human coxsackievirus A16 (PDB ID: 4jgy)^7^. The C-score for the best model was –0.61.

For VP1, empty human enterovirus (PDB ID: 3vbo)^26^ was provided as an external template in addition to the following templates selected by the programme: human enterovirus 71 (PDB ID: 3vbh)^26^, human enterovirus 71 (PDB ID: 3zfe)^33^, human coxsackievirus A16 (PDB ID: 4jgy)^7^, human enterovirus 71 (PDB ID: 4cdq)^34^, coxsackievirus A 9 (PDB ID: 1d4m)^32^, human rhinovirus 14 (PDB ID: 1ncq)^35^. The C-score for the best model was –0.75.

The homology models were rigidly-fitted into the HPeV3 map using the ‘fit-in-map' feature in UCSF-Chimera^36^. Using the ‘zoning' feature in UCSF-Chimera^36^, the HPeV3 capsid map was zoned to an asymmetric unit with a radius of 6 Å using the VP0-VP3-VP1 rigidly-fitted model. The VP0-VP3-VP1 model was flexibly fitted into the asymmetric unit using comparison of the results from two different flexible fitting programs iMODfit^37^ and FlexEM^38^ in order to arrive at a consensus fit^39^. Both these flexible fitting programs were used with the default settings. The models were then refined manually in Coot^40^ by truncating the models where no density was evident, improving the fits of main-chain and fitting the heavy side chains of the residues phenylalanine, tyrosine, tryptophan and arginine where visible. The models were further refined by real space refinement in Phenix^41^. All the visualization were carried out in UCSF-Chimera^36^.

A single copy of the RNA structured region was extracted using ‘Volume eraser' in UCSF-Chimera^36^. The extracted volume could accommodate about 30 nucleotides. A 30-nucleotide region (7181-7210) in the 3′-untranslated end of the HPeV3 isolate 152037 genome (GenBank ID: GQ183026) was folded on the RNAfold web server^42^ and the fold was modelled in RNAComposer^43^. This RNA model was fitted into the extracted volume using the ‘Fit-in-map' option in UCSF-Chimera^36^.

The footprint of AT12-015 was estimated by superimposing the HPeV3 atomic model into the HPeV3–Fab AT12-015 complex reconstruction and generating the roadmap for the atomic model with the fab footprint shown as contour lines in RIVEM^44^. All the amino acids residues which were surface-exposed within this footprint were taken as the epitopes for the Fab.

## Additional information

**Accession codes:** Density maps of HPeV3 icosahedrally-symmetric, HPeV3 asymmetric and HPeV3-Fab AT12-015 reconstructions have been deposited in the Electron Microscopy Data Bank under the accession codes EMD-3137, EMD-3322 and EMD-3138, respectively. The fitted models for HPeV3 have been deposited in the Protein Data bank in Europe with the PDB ID: 5APM. All the raw data collected for the HPeV3 reconstruction are available through the Electron Microscopy Pilot Image Archive under the accession code EMPIAR-10033.

**How to cite this article:** Shakeel, S. *et al*. Multiple capsid-stabilizing interactions revealed in a high-resolution structure of an emerging picornavirus causing neonatal sepsis. *Nat. Commun.* 7:11387 doi: 10.1038/ncomms11387 (2016).

## Supplementary Material

Supplementary Information Supplementary Figures 1-3 and Supplementary Table 1

Supplementary Movie 1 Fit of the models in the asymmetric unit of HPeV3 EM density map. VP0, VP1, VP3 models are shown in yellow, red and green, respectively, and their corresponding EM densities are shown as transparent surfaces in yellow, red and green, respectively.

Supplementary Movie 2 Fit of the RNA model in the asymmetric reconstruction of HPeV3 EM density map. The fitted-RNA model from Figure 2c was superimposed into one of the 60 RNA densities in the HPeV3 asymmetric reconstruction. The icosahedral symmetry copies were generated for this model in UCSF Chimera followed by zoning of the HPeV3 asymmetric reconstruction within 4 Å of these 60 symmetry-related RNA models. The RNA models are shown in magenta and the zoned EM densities are shown as transparent surfaces.
