# Supplementary material for: Multiple capsid-stabilizing interactions revealed in a high-resolution structure of an emerging picornavirus causing neonatal sepsis
Source: Nat Commun. 2016 Jul 20;7:11387. doi: 10.1038/ncomms11387 (PMC4961769; doi:10.1038/ncomms11387)
Supplement: Supplementary Information — Supplementary Figures 1-3 and Supplementary Table 1 [file ncomms11387-s1.pdf]

Supplementary Information

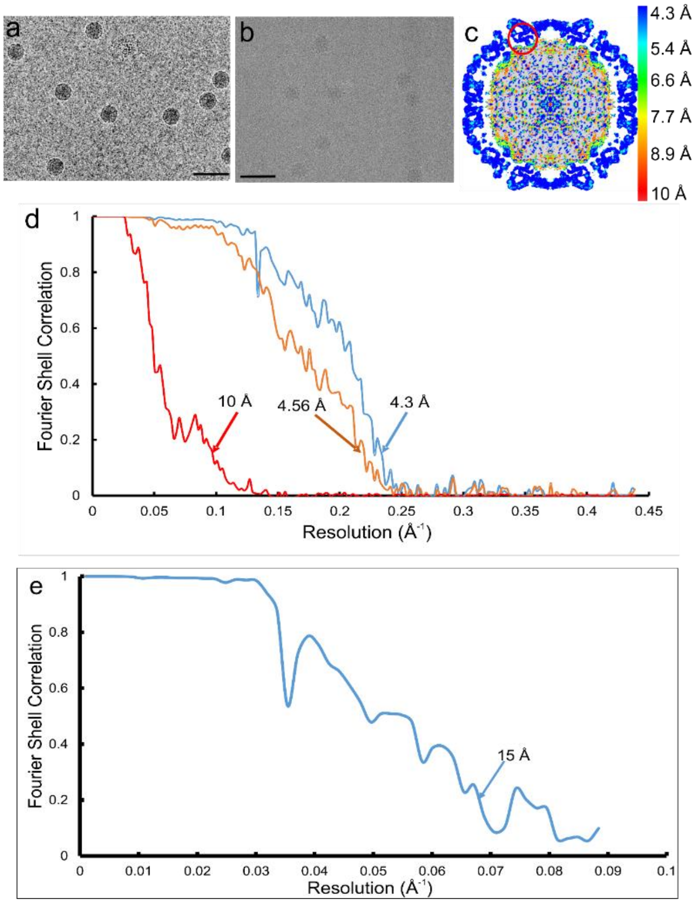

Supplementary figure 1. HPeV3 isolate 152037 microscopy and analysis. a) typical cryogenic electron micrograph of purified HPeV3 showing only filled capsids at a defocus of -2 µm. b) typical cryogenic electron micrograph of purified HPeV3 labelled with Fab showing only filled capsids at a defocus of -3 µm. (a and b) Scale bar 50 nm**.** c) Slab of the unfiltered HPeV3 reconstruction coloured according to the local resolution, estimated with ResMap. d) The gold-standard FSC 0.143-criterion plots from Relion for the two half-maps of the HPeV3 reconstruction without masking by 3D auto-refine option (orange), with masking by the post-processing option (blue) and without masking for the asymmetric reconstruction (red). e) Shows the gold-standard FSC 0.143-criterion plot for HPeV3-Fab AT12-015 reconstruction from AUTO3DEM.

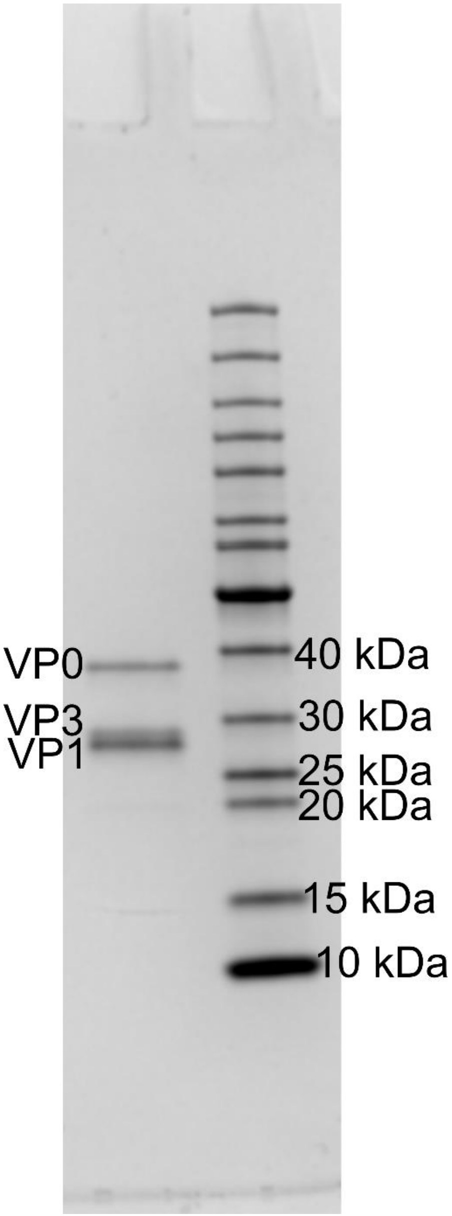

Supplementary figure 2. SDS PAGE. Purified HPeV3 isolate 152037 was run on a 4-20% tris-glycine gel under reducing and denaturing condition to show that the virions contain only VP0, VP3 and VP1 and no VP2 or VP4 were detected. A molecular weight marker is shown on the right.

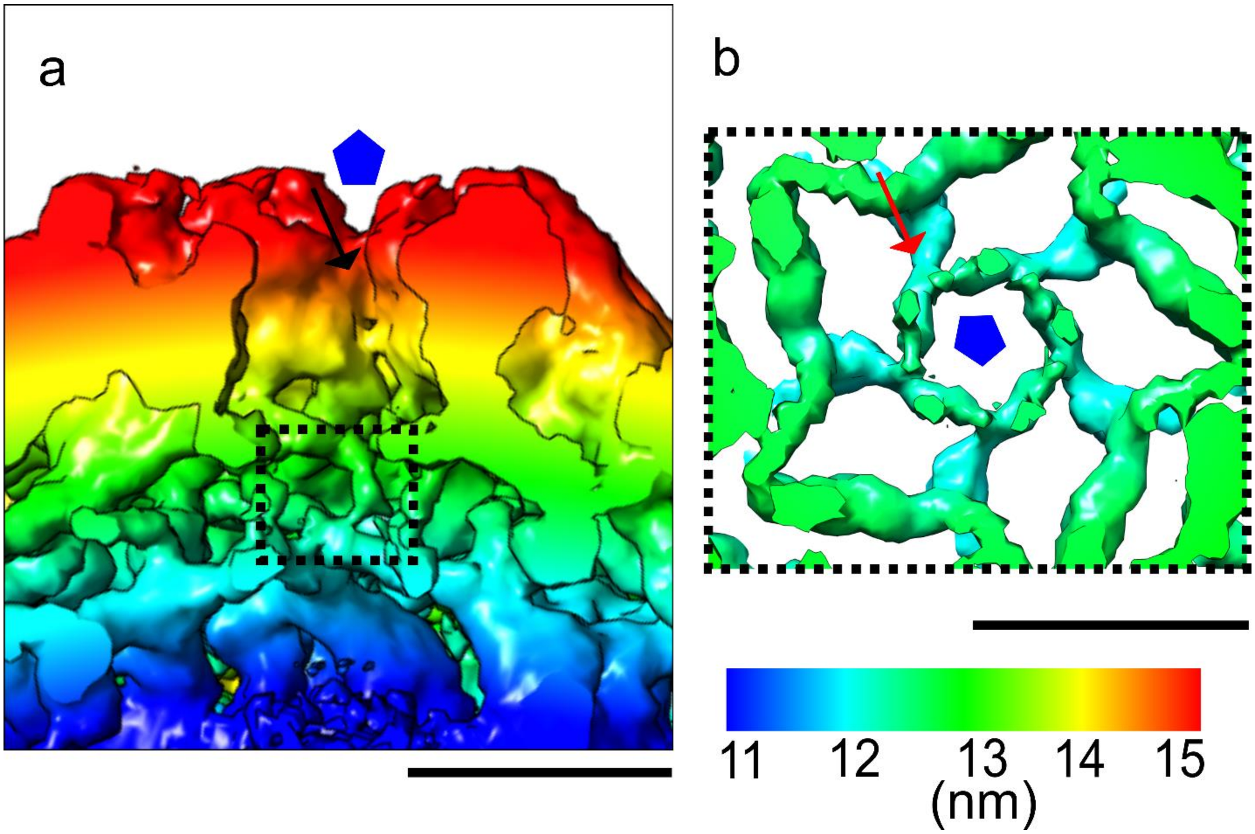

Supplementary figure 3. HPeV3 commonalities with other picornaviruses. a) Central cross-section of HPeV3 showing the open channel through the five-fold vertex (arrow). b) A 30-Å thick slab showing an annulus (arrow) below a five-fold vertex. Scale bar is 25 Å in both a) and b). The view in b) is orthogonal to a). The 5-fold symmetry axis is marked.

Supplementary table 1: Statistics of the reconstruction

**Parameter**	**HPeV3 icosahedrally-symmetric reconstruction**	**HPeV3 asymmetric reconstruction**	**HPeV3-Fab15 icosahedrally-symmetric reconstruction**
No. of particles used in the reconstruction	8889	41845	564
Underfocus range (µm)	0.42 - 2.34	0.42 - 2.34	1.46 - 3.56
Resolution (Å)	4.3	10.4	15
B-factor applied ($Å^2$)	-164.4	not applied	not applied
